# MEMS-in-the-lens architecture for a miniature high-NA laser scanning microscope

**DOI:** 10.1038/s41377-019-0167-5

**Published:** 2019-06-26

**Authors:** Tianbo Liu, Milind Rajadhyaksha, David L. Dickensheets

**Affiliations:** 1 Electrical and Computer Engineering Department, Montana State University, Bozeman, MT 59715 USA; 2 Dermatology Department, Memorial Sloan Kettering Cancer Center, New York, NY 10022 USA

**Keywords:** Confocal microscopy, Adaptive optics

## Abstract

Laser scanning microscopes can be miniaturized for in vivo imaging by substituting optical microelectromechanical system (MEMS) devices in place of larger components. The emergence of multifunctional active optical devices can support further miniaturization beyond direct component replacement because those active devices enable diffraction-limited performance using simpler optical system designs. In this paper, we propose a catadioptric microscope objective lens that features an integrated MEMS device for performing biaxial scanning, axial focus adjustment, and control of spherical aberration. The MEMS-in-the-lens architecture incorporates a reflective MEMS scanner between a low-numerical-aperture back lens group and an aplanatic hyperhemisphere front refractive element to support high-numerical-aperture imaging. We implemented this new optical system using a recently developed hybrid polymer/silicon MEMS three-dimensional scan mirror that features an annular aperture that allows it to be coaxially aligned within the objective lens without the need for a beam splitter. The optical performance of the active catadioptric system is simulated and imaging of hard targets and human cheek cells is demonstrated with a confocal microscope that is based on the new objective lens design.

## Microscopy: a new angle on miniaturization

Microscopes that generate images by laser scanning could be miniaturized using microelectromechanical systems (MEMS) to replace the larger components currently required, allowing more-effective imaging of small structures in laboratory animals and humans. Researchers in the USA led by David Dickensheets at Montana State University, developed the new concept using simulation studies and experiments to examine human cheek cells. The process uses a recently developed MEMS mirror to both scan and focus the laser beam. The mirror is part of a catadioptric lens, meaning one that builds its images using both the reflection and the refraction of light. This allows light to be gathered across a wide range of angles—technically described as a high numerical aperture—to generate the images. The system’s advantages are likely to allow improved diagnosis of medical conditions, including cancer.

## Introduction

Scanning laser confocal and multiphoton microscopy techniques are a mainstay for in vivo imaging of unprepared, uncleared organs in live animals^1–4^. Substantial progress has been made in imaging small animals, such as mice, that can be immobilized on the stage of a benchtop microscope^5,6^. Medical applications are also emerging. Large handheld or gantry-arm-mounted microscopes are used in dermatology clinics, which enable noninvasive and more thorough examination to reduce the dependence on physical biopsy for ruling out skin cancer^7–12^. However, the large size of a conventional laser scanning microscope limits its potential for both medical and live animal imaging. For imaging ambulatory animals and for accessing most of the human body, miniaturization of these instruments is necessary.

Miniaturization of the scanning mechanism was a necessary first step in the development of smaller instruments. Microelectromechanical system (MEMS) devices replace the bulky mechanisms that are required for scanning and focusing the beam with components that are only millimeters in dimension. This has enabled applications that were not previously possible. For example, a MEMS-scanned miniaturized two-photon microscope that weighed only 2.15 grams and was small enough to be mounted on the head of a freely moving mouse was used to image neuronal dendrites and spines within the brain^13,14^. MEMS has also facilitated the adaptation of laser scanning microscopy to endoscopic platforms^15–17^ and MEMS-based optical biopsy systems have demonstrated in vivo detection of cancer in regions of the head, neck, esophagus, and cervix^18–20^.

In addition to having a small footprint, a MEMS scanner contributes to miniaturization by combining multiple degrees of freedom into a single active element. A biaxial MEMS scanner replaces two bulky galvanometer scanners and, potentially, a lens relay between them. A 3D MEMS scanner can realize focus control in addition to 2D lateral scanning via a tip/tilt/piston motion^21–24^, tip/tilt/curvature control^25–27^, or a combination^28^. This eliminates the need for motor-driven mechanical focusing and further reduces the instrument size.

However, the instrument size depends on the optical architecture, in addition to the optomechanical components. One choice that can influence the size is whether to use preobjective scanning or postobjective scanning. The majority of MEMS-scanned microscopes (and fiber-scanned and fiber bundle systems) use preobjective scanning^29–36^, which requires an objective lens that is well corrected over a finite field of view. Miniaturized, high-numerical-aperture lenses that have been developed for this configuration require multielement designs with tight tolerances and long optical paths, which affect the instrument size^29,37–40^. On the other hand, postobjective scanning requires an objective lens that is corrected only for axial performance, which could be a single, small aspheric element. However, the deployment of a scan mirror after the objective lens requires a long working distance and, therefore, yields the best results if a small numerical aperture is used, which is appropriate for optical coherence tomography or dual-axis confocal imaging^41–46^. For single-axis confocal or multiphoton microscopy, higher NA (typically > 0.7) is required for adequate cross-sectioning and signal strength^47^. For these applications, postobjective scanning is impractical for a small device.

A third alternative is to deploy the MEMS scanner within the objective lens. A simple back lens, which is only required to operate on axis, generates a converging beam of modest NA that is incident on the MEMS scanner. The scanned beam passes through an aplanatic front lens, which increases the NA in the sample while preserving the diffraction-limited imaging over a finite field of view. This architecture has been adopted for both dual-axis confocal^18,20,48,49^ and fiber confocal fluorescence^22^ endoscopes with a hemisphere solid immersion lens as the front lens and tip/tilt/piston MEMS scanners. An aplanatic hemisphere increases the NA in the sample by *n*, which is the index of refraction of the glass; NA values of up to NA = 0.38 have been demonstrated^22^.

Here, we explore a new optical architecture for a miniature high-NA scanning laser microscope with a 3D MEMS scanner within the objective lens. We employ a folded annular beam, allowing the MEMS mirror to operate on axis. A low-NA back lens (only corrected on axis) illuminates the MEMS scanner. However, rather than a hemisphere solid immersion lens, we use an aplanatic hyperhemisphere front lens to increase the sample NA further. An aplanatic hyperhemisphere increases the NA by *n*^2^, thereby allowing our instrument to operate with NA = 0.7. The 3D MEMS scan mirror that we employ is of the tip/tilt/curvature type, namely, it integrates focusing with a deformable mirror surface to control the wavefront curvature^27^. Compared to previous mirrors of this class^25,26^, this mirror is capable of at least 2.5 times the focus stroke and demonstrates a *θD* product (a measure of the lateral resolution) of 12 deg-mm (0 to peak), which represents an improvement by a factor of 3.4 relative to the earlier mirror^25^. Furthermore, in this design, the 3D MEMS mirror has adaptive control for compensating spherical aberration throughout the 3D image volume, which becomes increasingly important for imaging at higher NA. The adaptive MEMS surface is a critical element in the overall objective lens design as it removes constraints on the glass elements and leads to a simple active optical system that preserves the diffraction-limited performance over a large 3D field of view.

**Fig. 1:**
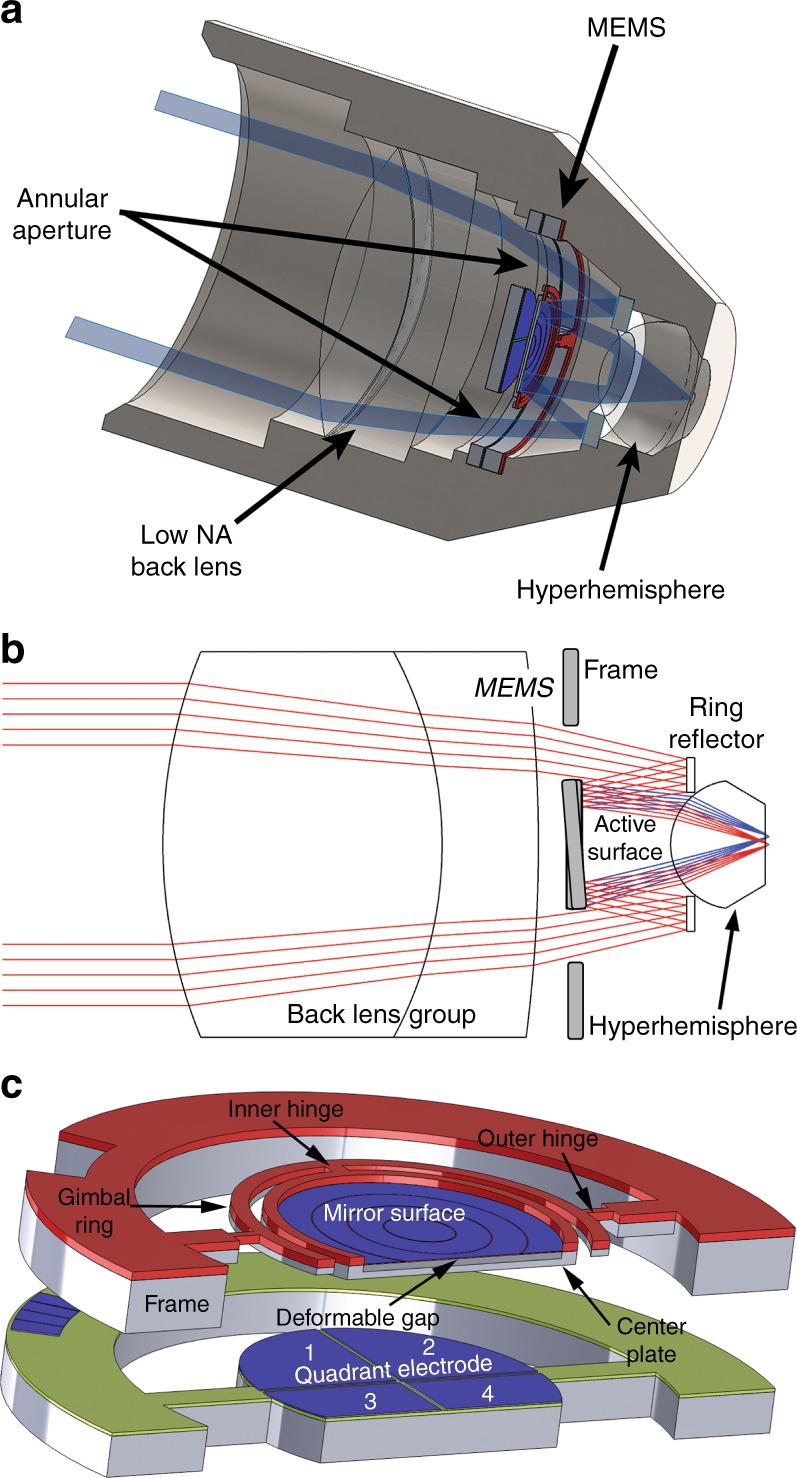
MEMS-in-the-lens architecture. **a** A cross-sectional view of the miniaturized confocal microscope with a new objective lens that incorporates a MEMS 3D scanner. **b** An illustration of the light path through the annular aperture and the beam scan of the MEMS device. **c** A model of the MEMS 3D scanner. A gimbal platform is bonded to a set of quadrant electrodes

The optical layout of the objective lens of the miniaturized confocal microscope is illustrated in Fig. 1a, b. By using an annular beam that passes through an aperture that surrounds the MEMS 3D scanner, the scan mirror can be integrated coaxially into the objective lens with the beam axis normal to the mirror surface without requiring a beam splitter to separate the incident and reflected beams. In our benchtop system (see Materials and Methods), we create the annular beam using a central stop, but it could be generated with greater optical efficiency by using, for example, axicon lenses or diffractive/holographic optical elements. The beam is focused by the relatively low-NA back lens group. The converging beam passes through the annular aperture that surrounds the MEMS mirror and is reflected by a ring reflector onto the active surface of the 3D MEMS scanner. The converging beam is scanned by the MEMS device onto the hyperhemisphere front lens, which increases the NA. Lateral translation of the beam focus is accomplished by tip and tilt motions of the mirror. Axial translation of the beam focus is accomplished by changing the curvature of the MEMS mirror. Spherical aberration is managed through fine control of the shape of the MEMS mirror.

## Results

### Simulation of the hyperhemisphere aplanat with active compensation of spherical aberration

The aplanatic hyperhemisphere front lens is ubiquitous in high-NA oil immersion objective lenses. With oil immersion, the hyperhemisphere can be exact, with an object distance from the lens surface (including a layer of index-matched oil) equal to the lens radius times 1 + 1/*n*. At this depth, the spherical aberration and circular coma of all orders disappear^50^, leading to diffraction-limited imaging over a large field of view. Focusing is performed by translation of the object, which changes the thickness of the oil layer. When imaging beneath the surface, the spherical aberration and coma begin to increase if the sample and the oil differ in terms of index of refraction.

**Fig. 2:**
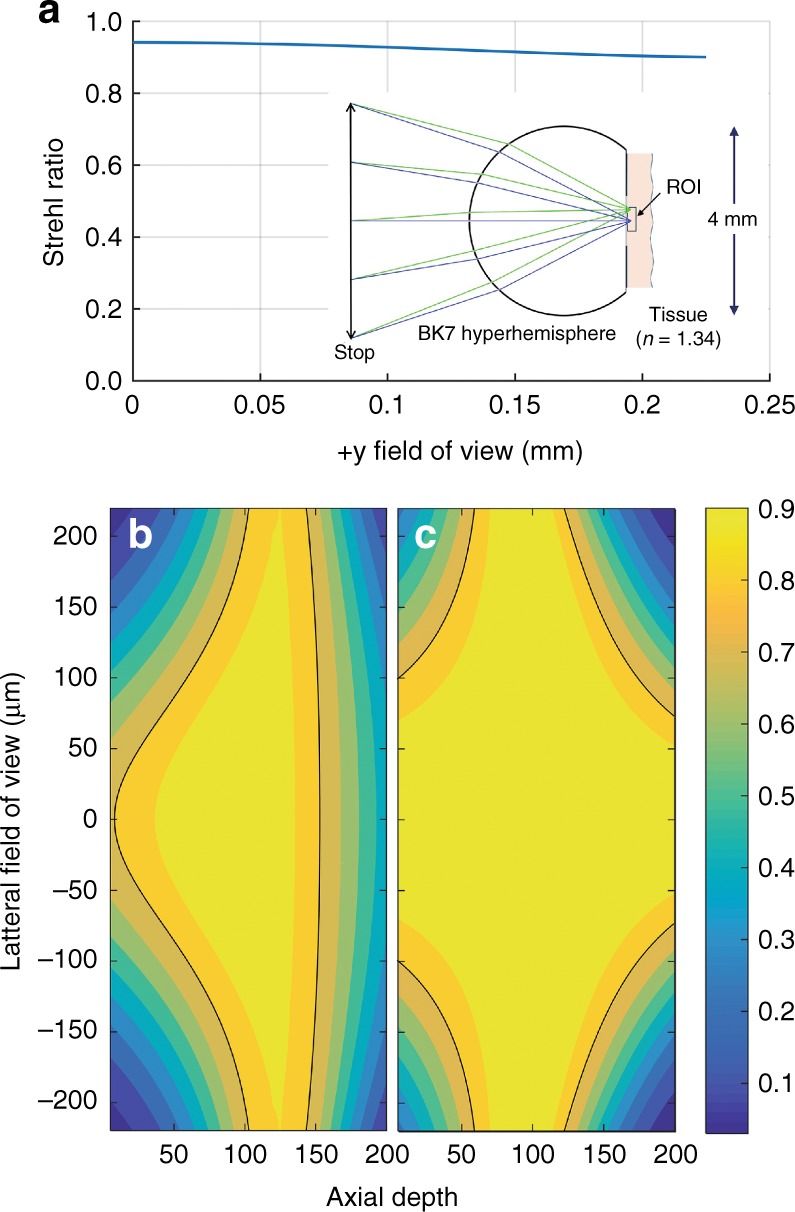
Simulated imaging performance. **a** A Zemax simulation of a 2-mm-radius BK-7 hyperhemisphere that is in contact with tissue. The aperture stop is 2.5 mm to the left of the lens, with NA = 0.7. **a** A plot of the Strehl ratio vs. the lateral field, which was simulated for a depth of 125 μm. **b**, **c** A contour plot of the Strehl ratio over a 2D axial cross-section of the 3D field of view. The black line represents the contour for *S* = 0.8. **b** Without depth-dependent adjustment of the spherical aberration. **c** With depth-dependent adjustment of the spherical aberration

For in vivo microscopy, we wish to operate with the hyperhemisphere in contact with tissue, which has a variable index of refraction in a typical range of 1.3–1.4^51,52^. For our simulation, we assume *n* for the tissue is constant with *n* = 1.34, which is close to that of water. We can compensate the spherical aberration at a specified depth (e.g., 125 μm) beneath the surface of the tissue by using a slightly thinner hyperhemisphere lens. The solution is not strictly aplanatic, but it performs well over a sizable field of view. Figure 2a shows the result of a full-aperture Zemax simulation for a 2 mm radius BK-7 hyperhemisphere lens that is 3.151 mm thick at a depth of 125 μm in the tissue. The NA is 0.7, the aperture stop is located 2.5 mm in front of the lens surface (this would be the position of a scan mirror), and we plot the Strehl ratio as a function of the field of view. The simulation wavelength is 633 nm. At this depth, the system is diffraction limited over a lateral field of view of > 450 μm (corresponding to a beam angle of ± 2 degrees).

Figure 2b presents a contour plot showing how the Strehl ratio varies over a depth of 0–200 µm with a lateral field of view of 450 µm. In this plot, the wavefront curvature at the aperture stop is varied to control the focal depth in the tissue, with no control over the spherical aberration of the beam. The wavefront sag at the aperture stop changes by 41.4 µm over the 200 µm depth range. Hence, a variable mirror sag of 20.7 µm is required to achieve a focus translation of 200 µm. According to this plot, the depth over which the lens is approximately aplanatic (and fully corrected over the full field of view) is fairly shallow. The dark contour corresponds to a Strehl ratio of 0.8, above which the system can be considered diffraction-limited. According to this metric, 43% of the volume of the cylindrical 450 × 200 µm 3D field of view is diffraction-limited, with an average Strehl ratio over the full volume of 0.63.

Figure 2c presents a similar contour plot of the Strehl ratio vs. the 3D field of view, except the spherical aberration is corrected at the aperture stop out to sixth order in the aperture radial variable, which provides a unique prescription for each depth in the simulation. This represents the effect of compensating the spherical aberration using dynamic surface shape control of the 3D MEMS mirror. In this case, the optimized hyperhemisphere thickness is 3.2 mm. For the full range of the focus depth, the coefficient values for wavefront correction of the primary spherical aberration $\left( {{\it{Z}}_4^0 = \sqrt 5 \left( {6\rho ^4 - 6\rho ^2 + 1} \right)} \right)$ ranged from −38 nm to + 127 nm (−0.06 to + 0.2 waves at 633 nm) and those for the secondary spherical aberration $\left( {Z_6^0 = \sqrt 7 \left( {20\rho ^6 - 30\rho ^4 + 12\rho ^2 - 1} \right)} \right)$ ranged from + 63 to + 253 nm ( + 0.1 to + 0.4 waves at 633 nm) using normalized Zernike polynomials as the basis. The percentage of the 3D field of view that is diffraction limited has increased to 69%, whereas the average Strehl ratio for the full field of view is 0.76. From this simulation, we conclude that a simple hyperhemisphere of BK-7 glass can be an effective front lens element for a tissue microscope with NA = 0.7 by using an active 3D MEMS scanner deployed at the location of the simulated aperture stop.

### Performance of the MEMS 3D beam scanner

The 3D scanner (Fig. 1c) is based on a dual-axis gimbal platform suspended using polymer SU-8 hinges. The dual-axis architecture enables tip-tilt scanning, which is actuated by a set of quadrant electrodes placed underneath the gimbal. Integrated onto the center plate is a large-stroke deformable mirror for focus control. The focus electrodes are concentric and enable the control of spherical aberration. The diameter of the active optical surface is 4 mm. An annular aperture is formed around the device to allow coaxial integration into the optical system. The mechanical properties of the mirror are summarized in Table 1^27^.

**Table 1:** Mechanical performance of the MEMS 3D scanner

Fast-axis resonant mechanical scan angle	± 3°
Fast-axis resonant frequency	1000 Hz
Slow-axis resonant frequency	~ 200 Hz
Slow-axis 1 Hz mechanical scan angle.	± 1.8°
Maximum mirror sag.	9.1 µm

Applying the Rayleigh criterion, the resolution of a circular mirror can be expressed as^53^ $N_r = 4\theta _mD/1.22\lambda _o$, where *N*_*r*_ is the number of resolvable spots, *θ*_*m*_ is the zero-to-peak mechanical scan angle, *D* is the diameter of the aperture (4 mm) and *λ*_*o*_ is the imaging wavelength (633 nm). At the maximum measured resonant fast-axis scan angle of ± 3° (*θ*_*m*_ = 3°), the mirror can resolve over 1080 spots. When operating at the ± 2° mechanical scan angle (*θ*_*m*_ = 2°) used for the Zemax simulations in the previous section, the mirror can achieve a resolution of *N*_*r*_ = 726 spots per line. The fast-axis resonant frequency of 1000 Hz allows imaging at four frames/second with a line density of 500 lines per frame using bidirectional scanning.

The range of axial focus is another crucial figure of merit for evaluating the performance of a confocal microscope. The mirror has demonstrated a deflection that exceeds 9 µm; when integrated into our optical system, this corresponds to 85 µm of focus range (*NA* = 0.7, *n* = 1.34). Previous membrane devices with similar construction have shown a 3 dB frequency response of ~ 2 KHz for focus control^54^. The deformable mirror has a measured correction range for $Z_4^0$ wavefront aberration from − 132 nm to + 228 nm (−0.21 to + 0.36 waves at 633 nm) and $Z_6^0$ from −178 nm to + 132 nm (− 0.28 to + 0.21 waves at 633 nm). This range is sufficient for performing the full correction for primary spherical aberration $Z_4^0$ and a portion of the correction for secondary spherical aberration $Z_6^0$ to yield the result presented in Fig. 2c. If necessary, the glass optics can also be designed to partially compensate with a fixed correction that would shift the correction range to coincide with the range that is provided by the MEMS device. In that case, the fully corrected range specified in Fig. 2c would be available.

### Demonstration of confocal imaging

**Fig. 3:**
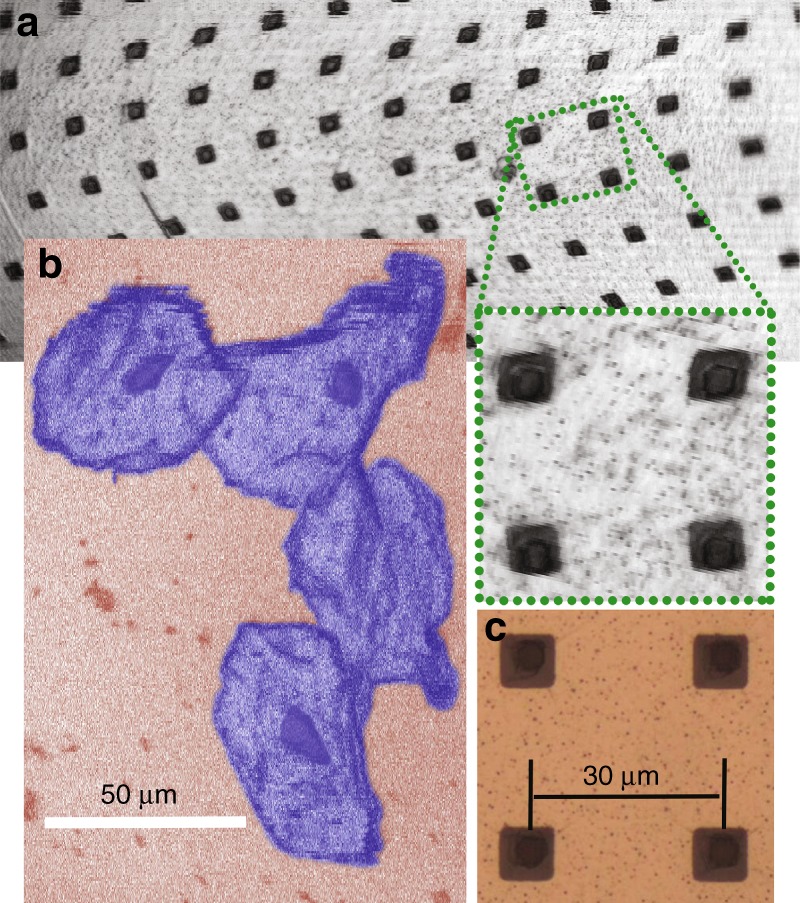
Experimental imaging results. **a** A confocal image of the surface of a prototype three-dimensional scanner. A subsection of the image is digitally enlarged to show details. **b** A confocal image of human cheek cells (with false coloring). The nucleus and cell membranes are clearly visible. **c** A digitally cropped brightfield epi-illumination microscope image of the surface of a similar prototype mirror that was recorded using a × 50 objective lens (NA = 0.8)

Confocal imaging was demonstrated using a benchtop mock-up of the new objective lens with an integrated 3D MEMS mirror. A three-dimensional (3D) MEMS scanner was used to provide 2D imaging and focus control for the microscope. No active control of the spherical aberration was employed for these figures. In the demonstration system, the object-space NA is 0.57, the pinhole NA is 0.06, and the pinhole diameter is 10 µm. Figure 3a presents a confocal image of a portion of a prototype scan mirror, which clearly displays details such as the release vias (holes) on the surface of the aluminum. The mirror was attached to the sample stage using a thin layer of water-based ultrasound gel. The etch-release vias on the surface of the mirror are arranged in a 30 μm square grid; the field of view of the system is ~ 390 µm by 180 µm. This corresponds to a ±1.6° mechanical angular scan along the fast axis (*y*) and ± 0.75° along the slow axis (*x*). A digitally enlarged subsection of the mirror surface image is also displayed. The dimensions of the openings in the aluminum are 7 µm by 7 µm. The dimensions of the holes that have been patterned into the underlying SU-8 membrane are 5 µm by 5 µm. The scattered specks are imperfections on the mirror surface, which may have resulted from contamination during the deposition of the aluminum thin film. These specks are also visible under a brightfield, epi-illumination microscope (× 50 magnification at NA = 0.8), as shown in Fig. 3c. The spatial arrangement of the vias follows a square grid pattern. However, according to the images, the linear grid appears to be slightly distorted. This could be owing to a misalignment of the electrodes underneath the gimbal, which pulls the mirror to one side at higher voltages and distorts the scan. This is more noticeable at the bottom of the frame, where the slow-axis voltage is the highest.

Figure 3b shows images of human cheek cells that were captured using the microscope. The cheek cells were introduced onto the sample stage using a cotton swab. A few drops of acetic acid (~ 6% concentration) in the form of balsamic vinegar were applied to the cells to enhance the nuclear reflectance. (It has been suggested that acetic acid induces alterations in protein structure in the nucleus^55,56^.) The size of the cheek cells is ~ 80 µm.

**Fig. 4:**
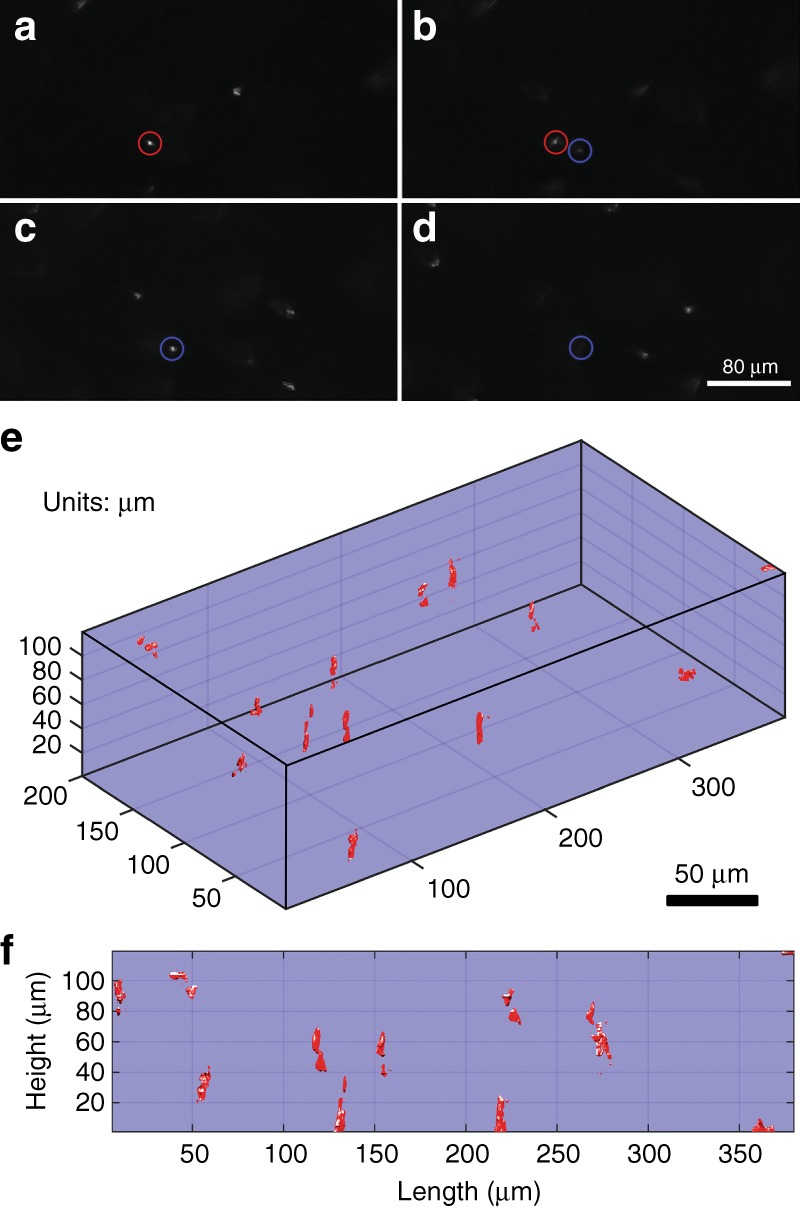
3D imaging demonstration. **a**–**d** Confocal sectioning of 6-µm-diameter polystyrene beads suspended in ultrasound gel. Two beads have been circled using different colors to show their focus change from frame to frame. **e** A volumetric reconstruction from the images recorded at each focal plane. **f** A first-angle projection through the volumetric rendering to better illustrate the confocal sectioning at different focal planes

The axial sectioning capability of confocal microscopes allows imaging beneath the surface of the sample. The 3D scanner was used to demonstrate this. For this experiment, a sample composed of 6 µm-diameter polystyrene microbeads suspended in ultrasound transmission gel $\left( {n_{gel} = 1.3} \right)$ was imaged. The initial focus of the system was positioned within the sample (~ 200 µm axially) so that the plane of imaging would remain in the sample as the beam focus was pulled toward the MEMS during the actuation of the deformable mirror. The applied focus voltage was from 0 V to 150 V, with the same applied voltage on all four membrane electrodes. The focus shift in the sample varies approximately linearly with the square of the applied voltage. Nonuniform voltage steps were used to maintain a relatively consistent *z*-step size during the 3D image acquisition. The total voltage-controlled focus range for the voltage range of 0–150 V was measured to be 127 µm in the gel sample. Figure 4 displays en face images of the beads at four different focus locations. A 20 µm-diameter pinhole was used for this experiment to improve the signal-to-noise ratio. Images a–d are each separated axially by 26 µm. Axial sectioning is clearly observed, with the in-focus bead from frame (a) (red circle) blurred in frame (b) and, finally, not visible in frames (c) and (d). Figure 4e displays a volumetric reconstruction of the beads from the image stack acquired by the MEMS confocal microscope. The first-angle projection through the volume is shown in Fig. 4f to better illustrate confocal sectioning at different focal planes. The reconstructions in Fig. 4e, f display isosurfaces of the intensities of the bead objects that correspond to a threshold that is set at half of the peak intensity of the object (50% intensity isosurfaces).

## Discussion

We described a new objective lens architecture that positions a 3D beam scanner between a high-NA aplanatic hyperhemisphere front element and a lower NA back lens group. The instrument features an annular aperture, allowing the scan mirror to be integrated into the objective lens, coaxially aligned to and normal to the optical axis without a beam splitter to separate the reflected beam from the incident beam. The back lens group can be a simple asphere as it needs only be well corrected for on axis spherical aberration (and, possibly, to provide chromatic correction for a fluorescence microscope). If the front lens is aplanatic (corrected for spherical and coma aberrations), then the performance of the integrated system can be diffraction limited over a wide field of view at depths where the aberration compensation is maintained.

The simulated performance of a 2 mm radius BK-7 hyperhemisphere aplanatic lens showed that the lens could be diffraction limited over 43% of a cylindrical 3D sample volume that was 450 μm in diameter and 200 μm deep, with the scanner only controlling the beam tip, tilt, and focus. However, with adaptive control over the spherical aberration, the imaging can be diffraction limited over 60% more volume, which corresponds to up to 69% of the full 3D sample volume. On axis, the correction is nearly perfect, and diffraction-limited performance was observed laterally over a finite field of view of 150 μm up to 200 μm depth. This will be useful for depth-resolved imaging for in vivo microscopy, for example.

Simulations were performed using a wavelength of 633 nm. For an optical pathlength aberration, the Strehl ratio improves for longer wavelengths because the optical pathlength error becomes less significant relative to the longer wavelength. On the other hand, using shorter illumination wavelengths, for example, for one-photon fluorescence excitation, the diffraction-limited volume will become slightly smaller, but the variation will be small across the visible spectrum. According to Maréchal’s formula, an RMS wavefront error resulting in a Strehl ratio of 0.9 at 633 nm would correspond to a Strehl ratio of 0.8 (and, therefore, still be considered “diffraction limited”) at 441 nm. At shorter wavelengths, light penetration into the tissue is diminished because of scattering, especially in heavily scattering tissues such as skin. In such cases, the system can be optimized for operation at shallower depths. On the other hand, at longer wavelengths, where there is less tissue scattering and the penetration depths can be higher, the fully corrected volume will be somewhat larger. For fluorescence microscopy, which requires correction at both the excitation and emission wavelengths, the back lens group could provide chromatic correction.

We developed a 3D MEMS mirror scanner that provides complete scanning and focus control for the instrument while also providing electronic control of the spherical aberration. The new 3D scan mirror demonstrates an improvement in the focus control range of 2.5 × and an improvement in the lateral scan resolution of 3.4 × compared with previously described 3D MEMS mirrors^25,26^. The mirror is provided with an annular aperture to allow its incorporation into a compact MEMS-in-the-lens system.

Finally, we built a mock-up of the proposed confocal system using our 3D MEMS scanner. We successfully used it to demonstrate imaging of a structured, high-reflectivity film, a sample of human cheek cells and confocal sectioning of suspended polystyrene beads. We clearly resolved submicron features in the highly reflective sample and showed confocal cross-sectioning when imaging the matrix of suspended beads. In the front projection image in Fig. 4f, whereas the beads are clearly resolved, the 3D profiles of each bead are neither uniform nor symmetric. This is a consequence of unavoidable interference effects (these are reflected light images, not fluorescence images) created by weak back-reflections from various uncoated glass surfaces; the symmetry may be further degraded by a small amount of astigmatism that was inadvertently introduced onto the MEMS scanner during manufacturing. Additional details are provided in the Materials and Methods section.

We have proposed a catadioptric MEMS-in-the-lens microscope objective lens that features an integrated MEMS 3D scanner for performing biaxial scanning and axial focus adjustment with dynamic control of spherical aberration. Based on our investigation, we believe the proposed instrument architecture shows considerable promise for future miniaturized high-NA laser scanning microscopes for in vivo imaging.

## Materials and methods

### Fabrication of the MEMS 3D scanner

The 3D scanner was constructed by bonding a micromachined gimbal platform with an integrated deformable mirror to a set of quadrant electrodes for tip-tilt actuation. The gimbal structure was fabricated with a silicon on insulator (SOI) wafer, whereas the electrode portion was fabricated using a double-side-polished silicon wafer. These two wafers will be referred to as the gimbal wafer and the electrode wafer, respectively. The gimbal platform features a silicon center plate that is supported by an outer silicon gimbal ring that is suspended via SU-8-based torsional hinges. Integrated onto the center plate is a deformable mirror that can be actuated using its own set of electrodes. The electrode wafer is of simpler construction: it carries a set of quadrant electrodes encapsulated in a dielectric polymer to prevent shorting in the event of incidental physical contact with the upper structure.

**Fig. 5:**
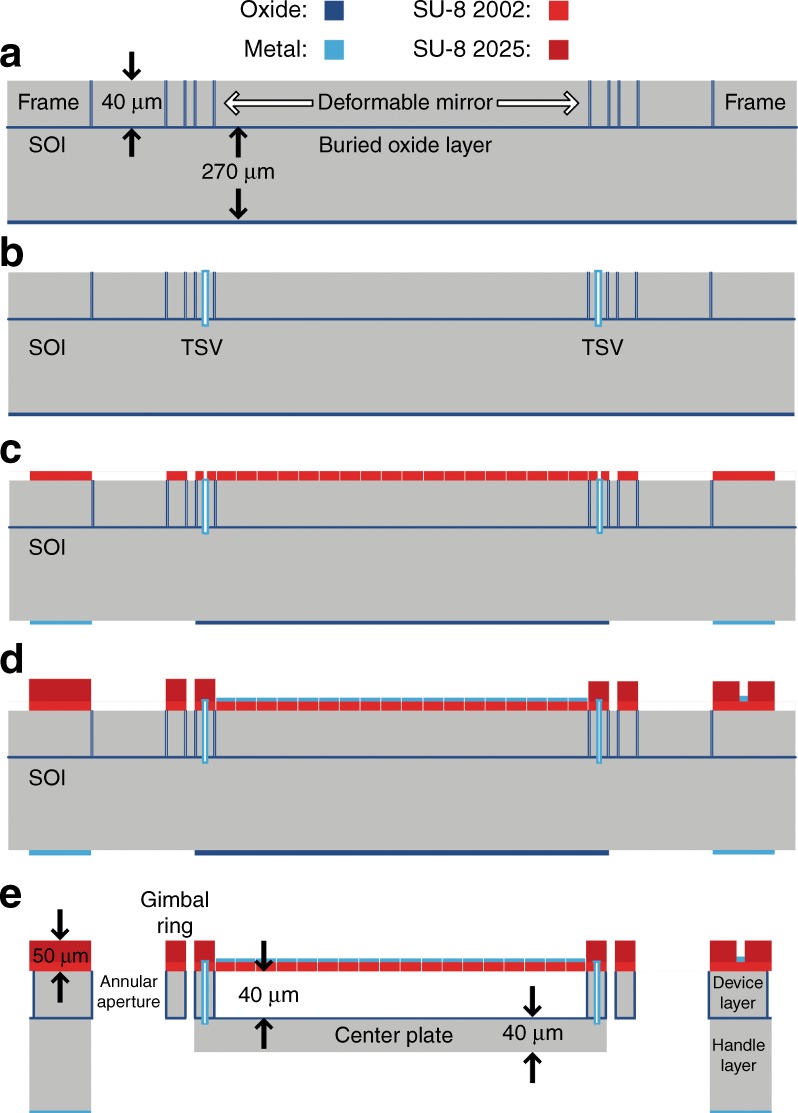
Gimbal wafer fabrication schematic diagram. **a** Etching, oxidizing, and patterning the vertical etch stops. **b** Creating TSVs. **c** Spin-coating and patterning the deformable membrane. **d** Depositing and patterning the top-side metal and spin-coating and patterning the SU-8 hinges. **e** Fully released gimbal platform

The fabrication process was described in detail by Liu et al.^27^. An overview of the process is provided here. The SOI gimbal wafer consists of device, handle and buried oxide layers with thicknesses 40 µm, 270 µm, and 300 nm, respectively. Figure 5 presents a schematic diagram of the fabrication process. Fabrication begins with vertical oxidized etch stops that are used during the release process to accurately define the dimensions of critical features. These etch stops were created by etching 3 µm wide trenches through the device-layer silicon to define the dimensions of the released features. Then, the wafer was oxidized and patterned to form the etch stops. The oxide on the backside of the wafer was also patterned during this step to create the first part of a bilayer differential etch mask that was used during the release step to define the thickness of the center gimbal plate. Next, through-silicon vias (TSVs) were formed to allow electrical connection from the surface of the SOI wafer to the handle-layer silicon, which would eventually become the center plate and act as the ground electrode for both scanning and varifocal actuation. To do this, vias were etched from the device-layer silicon past the buried oxide and into the handle-layer silicon. Then, they were coated with metal to provide electrical connection. The successful fabrication of these vias relied on a new and simple technique that uses progressive via sizing to mitigate the notching at the silicon-to-buried-oxide interface^27,57^. After the completion of the TSVs, aluminum was evaporated onto the backside of the wafer and patterned to form the second part of the bilayer differential etch mask. Next, the deformable membrane was constructed by spin-coating and patterning a 4 µm thick layer of SU-8. During this step, release vias on the surface of the membrane and ports were also formed to allow access to the TSVs. Then, a liftoff process was used to pattern a thin layer of aluminum (100 nm) as the optical surface of the deformable mirror. This reflective metallic surface was partitioned into four concentric rings to serve as the electrodes for varifocal actuation. The electrical bond pads on the frame of the device and the metal traces that route the electrical connection to the TSVs and concentric electrodes were also formed during this step. Then, thick polymer hinges and hinge anchors were formed by spin-coating and patterning a 46 µm thick layer of SU-8 2025. This layer, along with the previous 4 µm layer of thin SU-8, fulfills the designed thickness requirement of 50 µm for the SU-8 flexures.

**Fig. 6:**
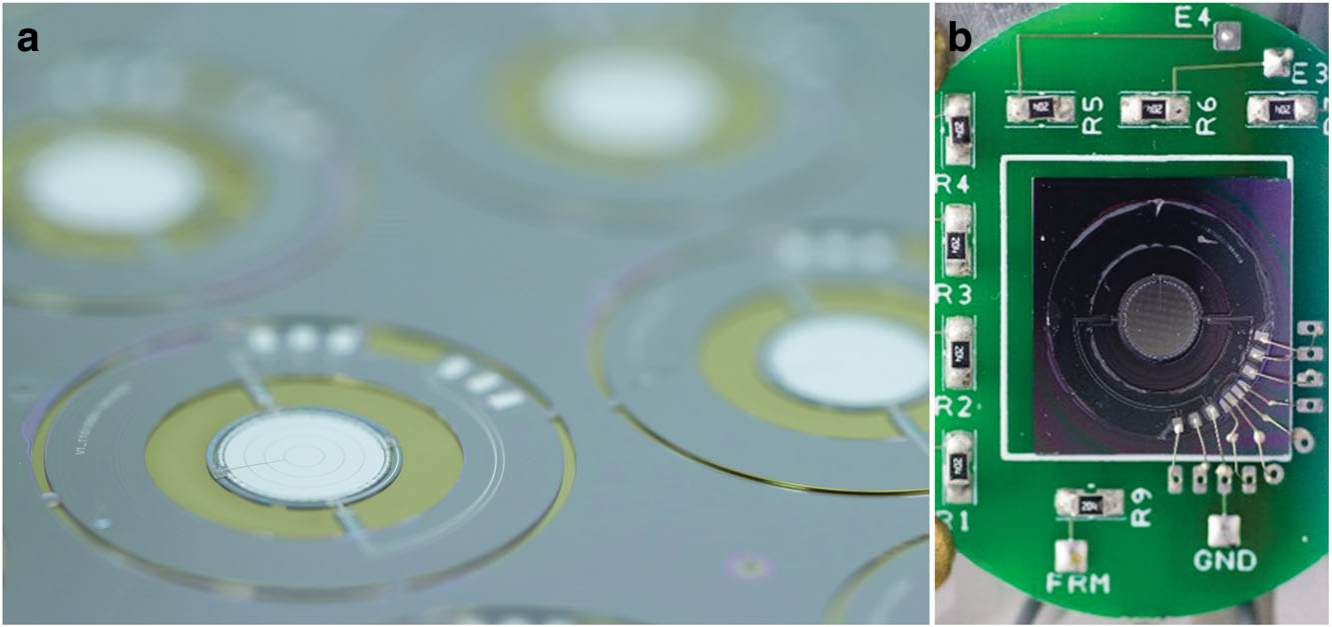
3D MEMS scan mirror. **a** Devices on wafer after the release process. **b** The MEMS scanner after wirebonding to support PCB

The release process begins with the use of the differential etch mask on the backside of the wafer to roughly define the thickness of the center plate. A silicon dry etch was used to create a step differential of ~ 50 µm. Then, dry oxide etching was performed to remove the oxide that covered the center gimbal plate. Next, the silicon etch was employed again to within 10 µm to 20 µm of the buried oxide. This remaining silicon was retained temporarily for structural integrity during subsequent steps. Then, xenon difluoride was applied to the top side only to remove the silicon from underneath the deformable mirror, thereby leaving it free-standing above the center plate. The silicon from between the gimbal structures and within the annular aperture was etched away simultaneously during this step. This xenon difluoride etch, although isotropic by nature, becomes guided and confined through the combined effects of the oxidized vertical etch stops, the buried oxide layer and the thin SU-8 film. Next, xenon difluoride was used to etch the backside of the wafer to clear the remaining silicon below the buried oxide. As the final step, a low-power dry oxide etch was used from the backside of the wafer to remove the exposed buried oxide. Figure 6a shows the devices on the wafer after the release process.

The fabrication of the electrode wafer was simpler. A double-side-polished wafer was oxidized, metallized, and patterned to form a set of quadrant electrodes and bond pads for electrical connection to external supporting printed circuit boards (PCBs). The design and fabrication processes of the electrode wafer accommodate the option of an annular aperture matching that of the gimbal platform. As this aperture does not affect the imaging results in this paper, it was omitted for ease of fabrication. A layer of SU-8 was spin-coated onto the electrode wafer to prevent shorting of the center plate to the quadrant electrodes in the unpredicted event of physical contact. Then, the singulated devices from the gimbal wafer and the electrode wafer were aligned and bonded. The completed 3D scan mirror was wire-bonded to a supporting PCB, which is shown in Fig. 6b.

### Focus depth and spherical aberration control

The 3D MEMS scanner adjusts the focus by electrostatically changing the curvature of the optical surface. The axial Rayleigh resolution, defined as the distance from the peak of the axial light distribution to the first null, can be expressed as $z_R = 2n\lambda _o/NA^2$, where *n* is the index of refraction of the medium being imaged (human skin: *n* = 1.34^51,52^), *λ*_*o*_ is the vacuum wavelength, and NA is the image-space numerical aperture. For the miniaturized microscope described in this paper, for NA = 0.70 and *λ*_*o*_ = 633 nm, *Z*_*R*_ = 3.46 µm. Based on paraxial Fourier analysis of a circular pupil, the phase delay that is required for shifting the focus by *z*_*R*_ is 2π*ρ*^2^, where *ρ* is a normalized radial variable at the pupil. A mirror sag of $\lambda _o/2$ is necessary to achieve this phase delay. Therefore, the number of axial zones (Rayleigh distances) that are resolvable can be expressed as $N_z = 2\delta /\lambda _o$, where *δ* is the maximum achievable mirror deflection. At higher NA, a derating factor *a* should be included such that $N_z = 2a\delta /\lambda _o$, with *a* = 0.86 for NA = 0.7. In this paper, the mirrors have demonstrated deflections that exceed 9 µm, which, when integrated into our optical system, corresponds to $N_z = 2 \times 0.86 \times 9/0.633 = 24.5$ resolvable zones. This provides 85 µm of focus range. This maximum deflection is currently limited by electrostatic pull-in. For our next generation of mirrors, a larger air gap under the membrane can further increase this focus range.

As described in the Results section, the spherical aberration of the system changes as a function of the focus depth. Therefore, the mirror was designed with concentric electrodes that add an additional degree of freedom for further tuning the optical surface to offset the induced spherical aberration as it is defocused. To vary the force radially on the deformable mirror, the concentric electrodes are biased using independent voltages. The amount of spherical aberration correction that can be achieved at a given defocus is limited by the differential voltage that can be tolerated between the electrodes, which is limited by electrical breakdown (arcing). The location on the 3D scanner that is most prone to electrical breakdown is at the hinges, where the separation between neighboring electrode traces becomes as small as 8 µm. To avoid arcing, it is necessary to evaluate the maximum voltage differential that can be tolerated. To do this, each of the concentric electrodes, in turn, were biased relative to the remaining three electrodes. The experimental results showed that the electrodes can handle differential voltages in excess of 200 V and up to 250 V between the outermost electrode and the innermost electrode. The increased tolerance between the outermost and innermost electrodes is owing to the layout of the electrodes and the traces on the device. Adhering to the voltage limitations that are specified above, the ranges of adjustment for first-order ($Z_4^0$) and second-order ($Z_6^0$) spherical aberration (coefficients of the normalized Zernike polynomials) were evaluated, which were superimposed on a baseline defocus ($Z_2^0$) value of ~ 1068 nm (3.7 μm nominal deflection). The range of observed values for the normalized Zernike coefficient of $Z_4^0$ was − 66 nm to + 114 nm for the mirror surface height, which corresponds to − 132 nm to + 228 nm of wavefront aberration. The range of observed values for the normalized Zernike coefficient of $Z_6^0$ was − 89 nm to + 66 nm for the mirror surface height, which corresponds to − 178 nm to + 132 nm of wavefront aberration. This does not represent the full range of values that can be achieved. A more in-depth analysis of the spherical aberration adjustment performance that can be realized using a deformable mirror that is similar to that of this paper was conducted by Lukes et al.^58^.

### Benchtop imaging demonstration

**Fig. 7:**
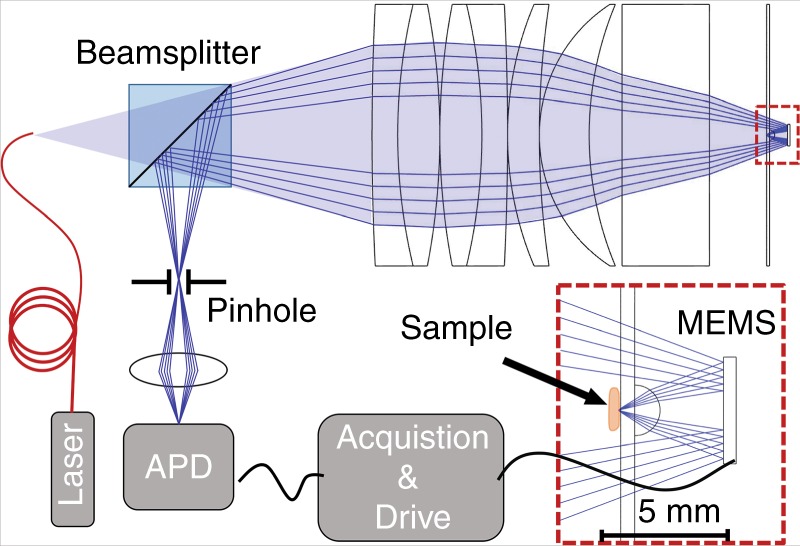
Schematic diagram of the confocal imaging setup. A magnified view of the MEMS scanner, hyperhemisphere, and sample stage is also included

A confocal microscope was constructed for assessing the imaging performance of the new objective lens with an integrated 3D MEMS mirror. A schematic diagram of the optical setup is shown in Fig. 7.

The benchtop system follows the compact optical system diagramed in Fig. 1b, except without the retro-reflection from an annular ring mirror. The beam is only reflected by the MEMS scanner, which can now be conveniently mounted at the right side of the setup. The system preserves the essential order with the sample, imaged by a hyperhemisphere lens, followed by the active 3D mirror scanner, as illustrated in the inset of Fig. 7. It also employs an annular beam. The observed performance is fully representative of the proposed MEMS-in-the-lens architecture. A consequence of the simpler optical test setup is that the sample is now located within the system, where it must be placed in contact with the glass using gel; the sample must also not exceed the diameter of the hyperhemisphere lens to prevent further obscuration of the imaging beam. However, this did not limit the field of view and this method of sample mounting did not negatively impact our experiments.

The illumination was from a 633 nm helium neon laser. The optical fiber was a single-mode fiber with NA between 0.10 and 0.14 and a mode field diameter of 3.6–5.3 µm (Thorlabs SM600 fiber). The objective lens system had an effective focal length of 14.78 mm (in air), an image-space NA of 0.57 and an object-space NA of 0.06, which was limited by the MEMS mirror as the aperture stop. The back compound lens group is also illustrated in Fig. 7. This lens group comprises two back-to-back achromatic doublets (Thorlabs AC508-200, *f* = 200 mm) followed by two meniscus lenses (Thorlabs LE1015, *f* = 200 mm and Thorlabs LE1076, *f* = 100 mm), all of which are in contact. A thick glass plate (18 mm) provides spherical aberration compensation for the desired focus depth of the instrument. A 2 mm diameter hyperhemisphere front lens was constructed from a 2 mm diameter half-ball lens (BK-7 glass) centered on and cemented to a 500 µm thick, 50.8 mm diameter glass wafer (D263T ECO glass), which also serves as the sample stage. The sample was attached to the side of the glass wafer opposite to the hyperhemisphere lens. The resultant 1.5 mm thick glass hyperhemisphere with 1 mm radius of curvature has minimal spherical aberration when imaging at a depth of 110 µm in water.

The MEMS scanner was mounted onto a stage (not shown) with three degrees of translational freedom and two degrees of rotational freedom facilitating focus adjustment and alignment. A 50/50 beam splitter was situated between the optical fiber and the compound lens element to separate the reflected light. A 10 µm-diameter pinhole was positioned conjugate to the optical fiber to spatially filter the reflected light. An avalanche photodiode detector was used to collect the light. The image forming beam is an annular beam, with the central portion blocked by the hyperhemisphere lens and the sample during the forward passage through the transparent sample stage. During imaging, a raster scan pattern was used in which the slow axis was driven nonresonantly using a sawtooth waveform (*V*_*y*_), whereas the fast axis (*V*_*x*_) was driven at its resonant frequency using a sinusoidal waveform. To date, the polymer flexures have shown no effects of aging (no change in the resonant frequency) despite accumulating over one billion cycles for the fast axis (> 280 h of operation).

The control voltages that were applied to the quadrant electrodes (Fig. 1b) are as follows: *V*_1_ = *V*_DC_ + *V*_*x*_ + *V*_*y*_, *V*_2_ = *V*_DC_ − *V*_*x*_ + *V*_*y*_, *V*_3_ = *V*_DC_ − *V*_*x*_ − *V*_*y*_ and *V*_4_ = *V*_DC_ + *V*_*x*_ − *V*_*y*_. For Figs. 3a, b, 4, the applied voltages were *V*_*DC*_ = 300 V, *V*_*x*_ = 200 V pk–pk and *V*_*y*_ = 300 V pk–pk. Figure 3 was cropped for display. Linear interpolation has been applied to all of the confocal images that are displayed in the Results section to correct for the sinusoidal distortion of the fast scan.

**Fig. 8:**
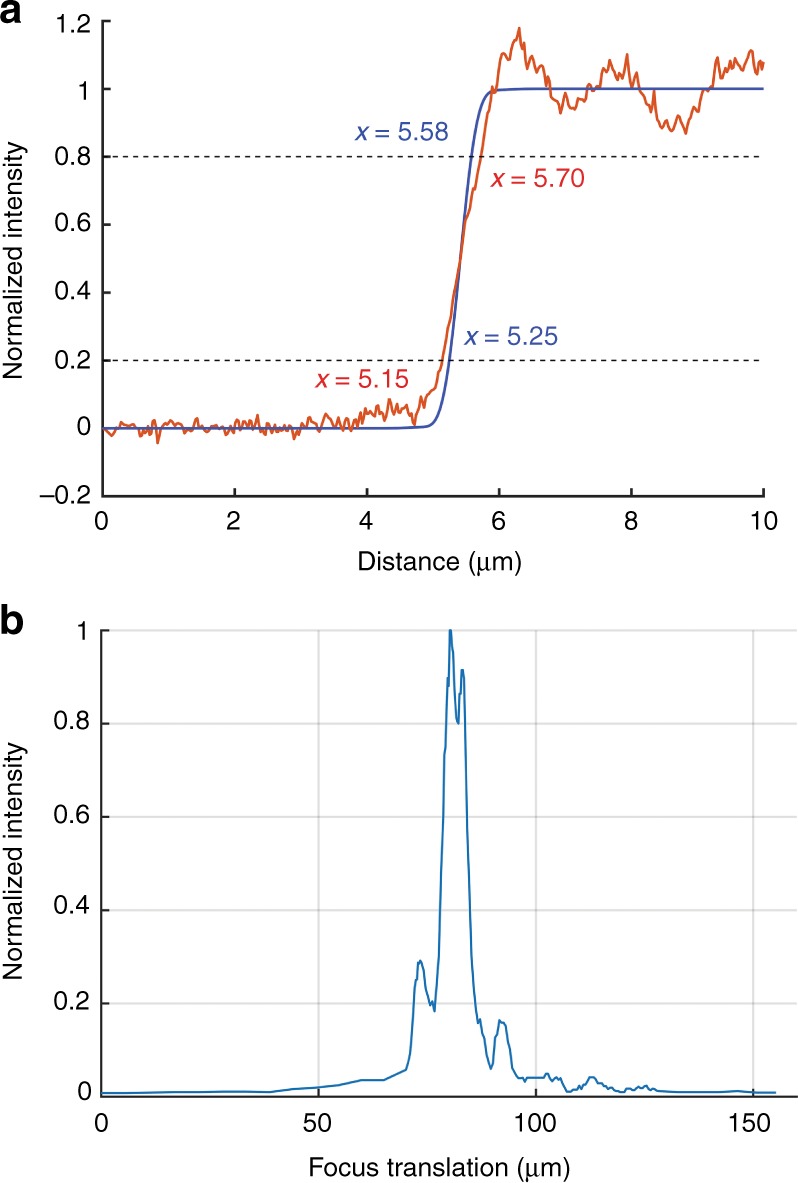
Lateral and axial resolution of bench-top system. **a** The edge response of the confocal system with 10 µm pinhole. The theoretical edge response is provided for comparison (blue). **b** The axial response of the optical system with 10 µm pinhole

The edge response was measured by using the MEMS scanner to image the edge of a cleaved wafer piece. A 65 µm thick microscope coverslip was inserted between the wafer piece and the sample stage to place the wafer edge near the imaging depth at which the spherical aberration is optimally compensated by the fixed optics. Figure 8a shows a plot of the intensity data as the beam was scanned across the edge. The distance was calibrated by imaging a target of known dimensions. The measured edge width from 20 to 80% intensity is 0.55 µm. This can be compared with the diffraction-limited 20–80% confocal edge width of 0.33 µm using NA = 0.57. We attribute the slightly degraded response to residual manufacturing error of the MEMS mirror. For the MEMS mirror that was used for this test, we measured residual aberration, which was primarily astigmatism, with an RMS surface variation of 42 nm across the 4 mm aperture with no voltage applied. This corresponds to an RMS wavefront error of 84 nm in the reflected beam. This RMS wavefront error (at a wavelength of 633 nm) is sufficient for degrading the Strehl ratio to ~ 0.5 and spreading the edge response, as we have observed.

To measure the axial response, a clean piece of silicon was mounted directly onto the sample stage (with no extra glass in the optical path). The axial focus position was adjusted by translating the MEMS mirror toward or away from the sample. For this measurement, the MEMS was acting purely as a mirror and was not deflected. The reflected light after passing through the pinhole was collected by the photodetector and the intensity was measured using an oscilloscope. Paraxial ray tracing was used to establish the relationship between the axial translation of the mirror and the axial translation of the focus point. The calibration was also verified experimentally using a microscope coverslip of known thickness (65 µm) with reflective markings on both sides. The markings on either side of the coverslip were imaged by moving the focus from the front to the back of the coverslip, requiring 115 µm of axial translation of the MEMS device. According to the paraxial ray trace, this equates to 63 µm of focus translation in the coverslip, which closely matches the actual thickness of 65 µm. The calibrated axial response of the system is shown in Fig. 8b. The results demonstrate a full-width-at-half-maximum value of 6.1 µm. The theoretical, aberration-free axial response, with a finite pinhole of 10 µm in diameter^59^, is 3.1 µm full width at half maximum. Similar to the edge response, the slight broadening of the axial response is consistent with the initial 42-nm RMS surface variation, which is mostly astigmatism, of the MEMS mirror that was used for the demonstration.

## Supplementary information

Supplemental Material
