# Supplementary material for: MEMS-in-the-lens architecture for a miniature high-NA laser scanning microscope
Source: Light Sci Appl. 2019 Jun 26;8:59. doi: 10.1038/s41377-019-0167-5 (PMC6592906; doi:10.1038/s41377-019-0167-5)
Supplement: Supplementary file 1 — Supplemental Material [file 41377_2019_167_MOESM1_ESM.docx]

Supplementary Material for

**MEMS-in-the-Lens Architecture for a Miniature High-NA Laser Scanning Microscope**

Tianbo Liu,^1^ Milind Rajadhyaksha,^2^ David L. Dickensheets^1*^

[tianbo.liu@yahoo.com](mailto:tianbo.liu@yahoo.com), rajadmil@gmail.com, davidd@msu.montana.edu

1. Electrical and Computer Engineering Department, Montana State University, Bozeman, Montana, 59715, USA.

2. Dermatology Department, Memorial Sloan Kettering Cancer Center, New York, New York 10022, USA.

*Corresponding author: [davidd@montana.edu](mailto:davidd@montana.edu), Tel: +1(406)994-7874, Fax: +1(406)994-5958.

**Linearization of the Sinusoidal Fast Axis Scan**

Linear interpolation has been applied to all of the images in the Results section to correct for the sinusoidal distortion of the fast scan. The slow scan is approximately linear and therefore did not require additional processing. However, the fast scan (*y*) is sinusoidal and requires linearization. The coordinates for each of the sample points in the direction of the fast scan can be calculated using the following:

$$y_{n}=A \cos(\frac{2\pi}{S_{c}}s_{n}), (1)$$

where $S_{c}$ is the total number of samples per cycle of fast scan and $s_{n}$ is the sample of interest. Assuming operation at 1000 Hz with a sample rate of 25 MS/sec, the total number of samples per cycle becomes 25000 samples (12500 samples per line), and equation (1) becomes:

$$y_{n}= A\cos(\frac{2\pi}{25000}s_{n}). (2)$$

Linear interpolation is then used to derive the intensity values at these coordinates. The sample processing and linear interpolation to form the image is performed in Matlab. The Matlab code is provided below.

%sinusoidal distortion correction for a set of images

%import .fig files into Matlab.

files = 'C:\Users\W97D925\Desktop\sine_correction\batch_corrections\test_files';

imags = dir(fullfile(files,'*.fig'));

%writing figure data into .m file.

for n = 1:size(imags);

open(fullfile(files,imags(n).name));

D = get(gca,'Children');

data = get(D,'CData');

close Figure 1

%process data (make x,y,I pairs for each pixel); The first number is

%samples per line and second number is lines per frame. This will vary

%according to the resonant frequency of the fast axis.

[Xlin,Ylin] = meshgrid( 1:12500 , 1:480 ); %x then y

%get Y locations

Y = Ylin;

%get x locations

radfreq = (2*pi)/25000; %spatial frequency of scan

cosreference = cos(radfreq*Xlin); %possible shift (need xlin-1 to be correct

X = ((-cosreference + 1)/2)*12500; %rescale so we still go from 1-12500 ish

%interpolate

Z = interp2(X,Y,data,Xlin,Ylin);

%plot

s = figure;colormap gray;

% clims = [0.046 0.068];

clims = [0.0 0.10];

imagesc(Z,clims);

daspect([14 1 1])

% saveas(gcf,'Barchart.png')

saveas(s,sprintf('Focus_position_b%d.png',n));

close all

end
